# Examples of Systemic Solutions for Skill-Mix Issues—An Analysis of International Experiences with a Focus on Rural Areas

**DOI:** 10.3390/healthcare14111501

**Published:** 2026-05-28

**Authors:** Jan Łuczak, Katarzyna Bochniak, Wiktoria Kaczmarek, Michał Szabelski, Zuzanna Staniaszek, Jakub Magdziarz Ibrahim-El-Nur, Magdalena Łoś, Aneta Nitsch-Osuch

**Affiliations:** 1 Faculty of Medicine, Medical University of Warsaw, Żwirki i Wigury 61, 02-091 Warsaw, Poland; janek1luczak@gmail.com (J.Ł.); wiktoria.kaczmarek2004@op.pl (W.K.); michal.szabelskii@gmail.com (M.S.); zuzannastaniaszek04@onet.pl (Z.S.); 2 Department of Social Medicine and Public Health, Medical University of Warsaw, Pawińskiego 3a, 02-106 Warsaw, Poland; jakub.magdziarz.20@gmail.com (J.M.I.-E.-N.); magdalena.bogdan@wum.edu.pl (M.Ł.); aneta.nitsch-osuch@wum.edu.pl (A.N.-O.)

**Keywords:** skill mix, task shifting, task sharing, healthcare workforce, primary care, rural health, medical deserts, multidisciplinary teams

## Abstract

**Background/Objectives:** Health systems in many countries are increasingly turning to task shifting, task sharing and broader skill-mix solutions to address staff shortages and unequal access to care, particularly in rural regions. This article summarizes how different healthcare systems introduce intersecting competencies through new professional roles, the expansion of the scope of existing professions, and the transfer of selected tasks between them. The aim was also to indicate which strategies may be relevant for the Polish context. **Methods:** A literature review was carried out using PubMed and Embase (14 February–9 March 2025). Publications from 2010 to 2024 in English or Polish were included. The study was designed as a narrative review supported by a structured literature search. Publications and policy documents relevant to skill-mix strategies were identified through a concept-driven process and incorporated into a thematic synthesis. **Results:** Countries that introduced new professional roles reported better access to care and more continuity for patients. Expanding the scope of nurses, physiotherapists and pharmacists often helped reduce the workload of doctors and, in some places, also supported better treatment results or smoother work in facilities. At the same time, there were clear challenges: unclear role boundaries, gaps in training, extra workload and limited organizational support. These issues were particularly visible in rural areas, where staff shortages are the most severe. **Conclusions:** Skill-mix strategies can support healthcare systems by improving access and the overall quality of care. However, their success depends on clear regulations, adequate preparation and acceptance among health workers. Although many solutions are promising, further research is needed to better assess their long-term effects and usefulness in medical deserts.

## 1. Background

The healthcare sector plays a crucial role in the functioning of society by ensuring access to medical care and promoting public health in urban as well as rural areas. The latter often face limited access to healthcare in many countries. A key element is the maintenance of a stable and efficiently operating system that supports the work of both medical and paramedical professionals. The effectiveness of this system largely depends on smooth collaboration between healthcare and allied personnel, a factor that gains importance in the face of challenges such as workforce shortages, an aging population, and a growing demand for healthcare services. The degree of cooperation among healthcare system personnel directly impacts the quality of services provided.

Historically, healthcare models were predominantly physician-centric. Decision-making was the exclusive domain of doctors, who were usually positioned at the top of the hierarchical structure. However, over recent decades, the above-mentioned new challenges of growing demand for healthcare services have forced a reevaluation of this paradigm. Healthcare systems are currently pressured to find a new balance between the accessibility of healthcare and financial sustainability, thus driving a shift towards more interdisciplinary teamwork without compromising the quality of care. Consequently, modern workforce management concepts in healthcare have evolved into political strategies aimed at mitigating personnel shortages while simultaneously improving the system’s resilience overall.

One way to optimize human resources in healthcare is through the skill-mix concept. This refers to a human resources management strategy in healthcare that involves adjusting professional roles through modification, expansion, or substitution. It may include transferring certain responsibilities from one medical profession to another, introducing new positions with clearly defined competencies, or making better use of the specialized knowledge of specific professional groups [1]. Combined with the idea of cross-cutting competencies, this approach enables more flexible use of personnel and improved service accessibility. These strategies are being implemented across various healthcare systems and constitute an important part of reforms aimed at enhancing the efficiency of the medical sector and combating the problem of medical deserts—regions with insufficient access to healthcare.

For the purposes of this review, it is necessary to clarify the terminology used in the area of human resources reorganization in healthcare. In this work, the term “skill mix” is used as an overarching concept referring to various forms of redistribution and optimization of competencies within medical teams. It includes, among others, task shifting (delegation of specific tasks from one professional group to another), task sharing (sharing clinical responsibilities among different professional groups), and the expansion or creation of new professional roles [2]. Although these terms are sometimes used interchangeably in the literature, they refer to distinct mechanisms within a broader strategy of workforce transformation.

The concepts of “rural areas” and “medical deserts” also require clarification. Definitions of rural areas differ across countries and may be based on criteria such as population density, geographic remoteness from urban centers, or the availability of public services [3]. In this paper, rural areas are understood as regions with lower population density and limited access to specialized healthcare services.

The term “medical deserts” refers to areas affected by persistent shortages of medical personnel and structural barriers preventing access to basic healthcare services [4]. These shortages may pertain to primary care, specialized care, or diagnostic infrastructure and are typically long-term in nature. In the present analysis, this term refers to systemic and entrenched disparities in the distribution of human resources rather than to temporary staff shortages.

The objective of this conceptually driven narrative review was to evaluate the solutions implemented in various healthcare systems internationally, with particular emphasis on cooperation between medical professions and strategies that leverage intersecting competencies, specifically within rural areas that meet the criteria of medical deserts. Crucially, the review aims to identify established skill-mix strategies and models that can serve as a blueprint for policymakers, especially in regions facing the crisis of medical deserts. Furthermore, the study intends to describe the potential risks and advantages of implementing skill-mix solutions, thereby preparing policymakers for potential systemic consequences.

While effective rural workforce policies are usually multi-component, this review focuses on skill mix as a distinct category of interventions. To better understand the potential effects of skill-mix interventions, it is useful to present a simplified theory-of-change model explaining the mechanisms through which these strategies may influence the functioning of the healthcare system. In the literature, changes in the structure of competencies (skill-mix change) are described as part of a broader reorganization of medical teams and service delivery models [5].

Interventions involving the introduction of new professional roles, the expansion of existing professions’ scopes of practice, and the delegation and sharing of tasks lead to a redistribution of clinical and administrative responsibilities within healthcare teams. The direct mechanism of action consists of a more efficient alignment of tasks with the competencies of specific professional groups, which may enable an increase in the volume of services provided, improved continuity of care, and better adaptation of services to patients’ needs [6]. In rural settings, such approaches often involve non-physician and non-traditionally trained cadres, supported by targeted training and supervision.

In the short term, this may result in reduced waiting times, improved access, and higher patient satisfaction, although the magnitude of these effects depends on the implementation context. Achieving long-term systemic outcomes—such as workforce stabilization, reduction in territorial inequalities, or sustained improvements in cost-effectiveness—depends on additional institutional and regulatory factors [7]. These include, among others, clear legal frameworks, adequate funding, appropriate training and supervision, organizational support, and coherent workforce planning policies.

It should be emphasized that skill-mix interventions alone do not eliminate the structural causes of medical deserts, such as chronic underfunding, infrastructure shortages, or adverse demographic trends. Their effectiveness is conditional and depends on the complementary implementation of broader systemic reforms.

## 2. Materials and Methods

### 2.1. Study Design

This study was designed as a narrative review with a structured literature search, aiming to provide a conceptual and system-level analysis of skill-mix strategies and overlapping competencies among healthcare professions. The review focused on identifying models of task redistribution, the expansion of professional roles, and organizational solutions intended to optimize healthcare workforce capacity and reduce pressure on selected professional groups.

### 2.2. Literature Identification and Structured Database Search

The structured literature search was performed in two electronic databases: PubMed and Embase. The search process was conducted during the period from 14 February 2025 to 9 March 2025, using several predefined keyword combinations related to workforce organization and professional roles. These keyword combinations were applied separately in each database, including:

- “primary care” AND “skill mix”;
- “nurse” AND “skill mix”;
- “physiotherapist” AND “task shifting”;
- “dentist” AND “skill mix”;
- “pharmacist care” AND “risk factors”.

Each keyword combination was intended to capture literature relevant to a specific healthcare profession or care setting within the broader concept of skill mix.

The search was limited to publications written in English or Polish and published between 2010 and 2024 to include only complete calendar years in the analysis. The purpose of the structured search was to enhance transparency and consistency in the literature identification rather than to establish an exhaustive dataset for systematic synthesis.

PubMed and Embase were selected as the primary bibliographic databases because they provide a comprehensive foundation of peer-reviewed research on healthcare workforce organization. However, to capture system-level and organizational perspectives not consistently indexed in bibliographic databases, the search was complemented by gray literature. This included legal and regulatory acts, OECD reports, and institutional guidance documents issued by international and national health authorities, such as the WHO, the OECD, the Polish Ministry of Health and the National Health Fund. To ensure full transparency regarding the gray literature search strategy, including specific website domains, search terms, and dates, a detailed summary is provided in Appendix A.

### 2.3. Selection of Publications

**Figure 1:**
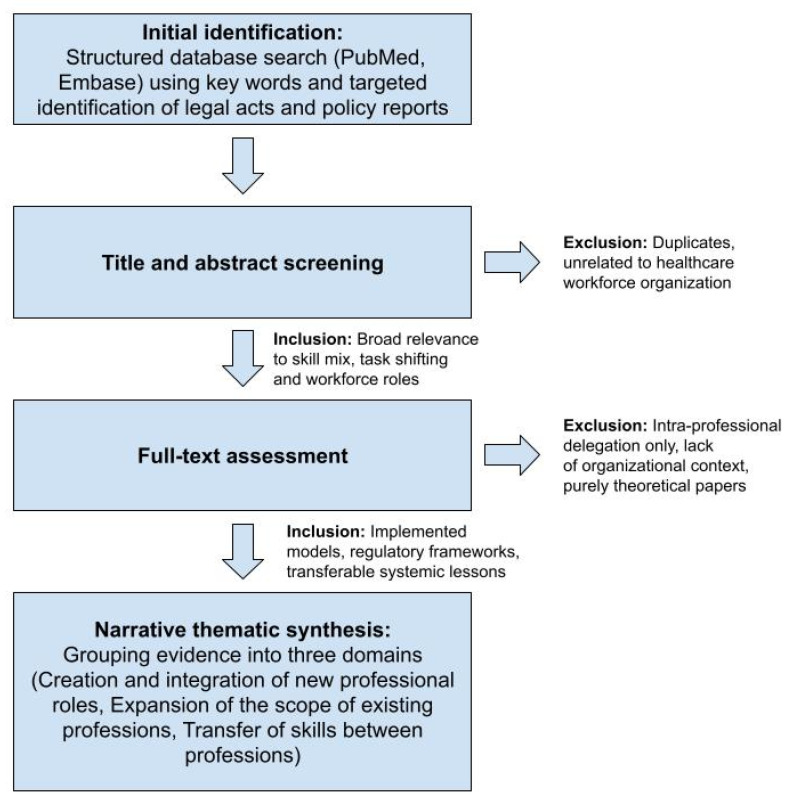
Conceptual selection and synthesis framework.

Following initial identification, the selection process underwent two distinct phases to ensure methodological transparency, as visually summarized in Figure 1. The entire procedure was iterative and concept-driven, focusing on the quality and relevance of evidence rather than rigid systematic thresholds. In the first phase, which involved title and abstract screening, five reviewers assessed publications for broad thematic relevance to skill mix, task shifting, and the development of new professional roles. At this stage, exclusion criteria were applied to remove duplicate records and publications focusing on topics unrelated to healthcare workforce organization.

In the second phase, a more explicit, concept-driven assessment was conducted, in which full texts were evaluated based on their specific contribution to the analytical objectives of the review. At this stage, inclusion required documents to report implemented models, policy frameworks or transferable system lessons. Publications focusing exclusively on intra-professional task delegation or purely theoretical considerations without system-level implementation were not retained for the final synthesis. Disagreements regarding selection were resolved through collective discussion by the research team, and the final selection was agreed upon by consensus.

In addition to publications identified through the structured database search, the final set of sources included a limited number of publications identified in parallel through an expert-led literature review conducted by multiple authors during the search period. These publications were retained using the same concept-driven criteria applied to database-derived records, based on their relevance to healthcare workforce organization and contribution to the analytical objectives of the review.

### 2.4. Data Extraction and Narrative Synthesis

Key information was extracted from selected publications, including the healthcare system context, professional groups involved, the type of skill-mix intervention, and reported organizational or system-level outcomes. The collected data were analyzed using a narrative thematic synthesis approach, applied iteratively to identify recurring concepts and system-level patterns. This process involved coding and grouping findings to identify common patterns across different skill-mix interventions. The results were then integrated with empirical evidence, policy documents, and regulatory frameworks to develop a coherent conceptual narrative, rather than to produce aggregated outcomes or quantitative comparisons. In order to avoid double-counting, when multiple documents described the same policy or intervention, we treated the specific country or healthcare program as a single case. We also applied a basic hierarchy of evidence, where legal acts and official documents provided a regulatory framework, while empirical studies and systematic reviews were used to assess actual outcomes and results. For the purpose of structuring the presentation of results, the narrative synthesis was organized around three overarching thematic domains:

1. Creation and integration of new professional roles;
2. Expansion of the scope of existing professions;
3. Transfer of skills between professions.

### 2.5. Methodological Considerations

Given the narrative nature of the review and the heterogeneity of included sources, no formal risk-of-bias assessment was performed. Source credibility, relevance, and clarity of reported interventions were considered during synthesis. This review did not aim to identify all available publications on the topic but rather to select a representative set of sources that were conceptually relevant at the system level.

To improve transparency and comparability across studies, a structured evidence summary was developed, which includes the following elements: author (year), country, setting, cadre(s) involved, skill-mix strategy, regulatory/financing context, outcomes reported, and study type.

## 3. Results

**Figure 2:**
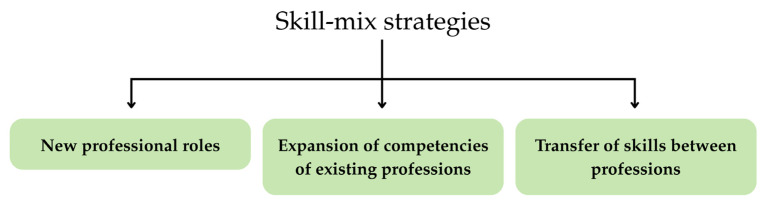
Key components of the skill-mix concept.

This review highlights three main areas within the intersecting competencies concept: the introduction of new professional roles, the expansion of competencies within existing professions, and the transfer of skills between professions (Figure 2). The results are presented according to the three main types of skill-mix interventions: the creation of new roles, the expansion of existing competencies, and the transfer of tasks between professions. Within these areas, we also identified recurring themes such as regulation, training, and professional acceptance, as well as common enabling factors and barriers. Rural and resource-limited settings are discussed in sections where they were directly addressed in the included studies.

### 3.1. Creation and Integration of New Professional Roles

The creation and integration of new professional roles into the healthcare system undoubtedly have certain advantages. According to Tsichristas A. et al., the introduction of the new professional role of “advanced nurse practitioner” led to increased access to healthcare for patients and an increase in the quality of care provided [6]. Consequently, better clinical outcomes and increased patient satisfaction and that of their relatives were noted. It was also observed that once the emerging role had adapted to the new workflow, healthcare costs decreased, meaning that the profitability of the medical facility increased.

The UK is an example of a country that is already implementing new professions in primary care. The National Health Service (NHS) has established the “ARRS” program, which provides limited funding for primary care facilities to hire additional non-medical staff, including clinical pharmacists, physician associates, physiotherapists and paramedics [7]. A survey of primary care managers in the UK indicates a clear upward trend in hiring staff under the “ARRS” program [8]. The most common motivators for hiring these staff members were an increase in the number of available appointments, a desire to relieve the burden on general practitioners (GPs), an improvement in the cost-effectiveness of the facility, and the desire to achieve a better match between the available medical team and patients’ needs. Primary care managers specified that the ideal medical team should include more non-medical staff, indicating a rising role for these new professions in the primary healthcare setting.

The introduction of new roles can also be associated with certain difficulties and consequences, especially at the initial stage of their introduction. As Nelson P.A. et al. noted, a lack of a detailed definition of new professional roles and the lack of specific descriptions of their competencies are among the primary challenges [9]. These deficiencies make it difficult to integrate emerging professions with existing medical staff. Some hostility on the part of GPs as well as nurses toward the new roles was also observed, perhaps related to the unfamiliarity of the situation and the fear that their duties would be taken over by the newly introduced professions. Those occupying the new roles, who had no previous experience with how primary healthcare works, declared that they were initially put off by the unpredictability and lack of standardization of primary healthcare work. Gaps in training and practice for new roles proved to be a frequent difficulty. New employees often need time and mentoring in order to fully carry out their assigned tasks.

The increasingly holistic approach in medicine and the expansion of the patient care workforce have resulted in the need to coordinate patient care. The function of a care coordinator may vary in different healthcare systems and may be performed by different medical professionals.

In Poland, many comprehensive patient care programs, such as POZ PLUS, have been established, resulting in the need for a new professional role of a healthcare coordinator [10,11]. The coordinator handles all administrative activities related to the patient. However, the exact scope of duties performed is determined by each individual primary care practice.

An important issue is determining who should be responsible for coordinating patient care. In Switzerland, the coordination of home palliative care is not systematized and is being performed by different medical professions as well as by family members of the patient. One study found that there is ambiguity present regarding who should play the most important role in coordinating palliative care [12]. This is due to the fact that in this setting, the role of a care coordinator is not a specifically defined position. Rather, a coordinating role is spontaneously assumed by people actively caring for the patient. The lack of a defined coordinator role leads to a situation where it is the patient’s family that has to coordinate the care of their loved ones. As a result, family members are overburdened and under-supported financially.

The creation of new professional roles is also being implemented within dental care. Many studies point to the benefits of dental assistants and dental hygienists. According to a UK-based study, procedures that can be delegated to dental therapists account for approximately 77.6% of dentists’ clinical time [13]. A different study additionally found that a larger number of dental assistants is associated with an increase in patient visits per week [14]. A study in Bulgaria found that EFDAs (Expanded Function Dental Auxiliaries), or dental auxiliaries, can improve practice efficiency [15]. The majority of Bulgarian dentists find it beneficial to expand and increase the skills of dental assistants.

In Scotland, as part of the national “Childsmile” health program, extended-duty training for dental nurses is provided [16]. The duties of the new assistant’s role include dietary advice, oral hygiene advice and fluoride varnish application. According to Wendy Gnich et al., trained dental assistants declared a high level of satisfaction regarding their new role, found the program’s training useful, and often used their newly acquired skills in everyday practice, delivering quality clinical preventive care [17].

Integrating new professional roles (such as advanced nurse practitioners, care coordinators, and allied health staff) is an important strategy for expanding healthcare accessibility in rural areas. Deploying these non-physician professionals into primary and dental care may significantly increase appointment availability and relieve the clinical burden on scarce GPs, thereby improving practice efficiency. While early implementation often faces organizational hurdles like undefined competencies, training gaps, and staff resistance, overcoming these challenges is essential. Well-mentored interdisciplinary teams prevent the overburdening of rural physicians and patients’ families, ensuring sustainable and equitable healthcare delivery in underserved regions.

### 3.2. Expanding Scope of Existing Professions

The expansion of competencies of existing medical professions is a vital element in developing a more effective healthcare system. Lightening the workload of GPs can be achieved by allowing other healthcare professionals to take over some of the duties previously performed only by physicians. In Poland, nurses and midwives gained the right to perform some emergency medical procedures and provide healthcare services [18]. Additionally, physiotherapists are now allowed to conduct patient visits independently [19]. These changes not only help to relieve the burden on doctors but also improve patients’ access to care.

Similar solutions have been implemented in other countries. In the Netherlands, care of older adults is divided among general practitioners, registered nurses, physician assistants and general nurses [20]. Thanks to the increase in competencies in these professions, doctors can concentrate on more complex cases, which leads to better resource management and improved treatment coordination within medical teams. Van Erp et al. report that integrating nurse specialists (and, to a lesser extent, physician assistants) into the “primary care plus” model allows for the delivery of care quality comparable to that provided in hospital outpatient settings, while at the same time potentially reducing the number of unnecessary referrals to secondary care [21]. The expansion of responsibilities for nurses and doctors’ assistants has also made these professions more attractive to prospective students. However, the lack of clear regulations regarding the scope of responsibilities and salary structures may hinder the successful implementation of such reforms. Furthermore, patients may be unaware of the newly assigned qualifications of registered nurses or doctors’ assistants, potentially causing uncertainty regarding the quality of services received.

In Taiwan, the skill-mix model is based on close collaboration between registered nurses and nurse assistants. Research has shown that introducing nurse assistants and reorganizing nursing duties can reduce the operational costs of healthcare centers [22]. Thanks to the support from their assistants, nurses are able to spend more time with patients and their families, performing a more educational and supervisory role.

An article published in 2024 showed that expanding the scope of nurses’ responsibilities in primary healthcare can maintain, and in some indicators even improve, clinical outcomes while ensuring high patient satisfaction [23]. However, the authors emphasize that these benefits depend on the nurses’ qualifications and highlight the need for better documentation of training and working conditions.

Expanding the competencies of pharmacists is an example of successful task shifting. There is evidence of a beneficial impact of expanding the competencies of pharmacists on patients’ lifestyle modifications as well as improved patient compliance. According to reports from multiple countries, greater involvement of pharmacists in outpatient care has led to a reduction in cardiovascular risk among patients [24,25,26]. Pharmacists can also share their expertise in the field of drug interactions, adverse effects and dosages with other healthcare workers. They are able to implement their own recommendations on how patients’ treatment should be modified [27]. Pharmacists also had a beneficial influence on oncology treatment in Nicosia by enhancing patients’ awareness of their health condition and reducing their concerns related to the adverse effects of chemotherapy [28]. As a result, patients became more aware of the importance of following medical recommendations, which could contribute to achieving better treatment results. Furthermore, during a pilot study conducted by the NHS, over 400 pharmacists were employed in local general practices, while their competencies were expanded [29]. The aim of the pilot study was to improve patient care, expand primary care staff, and ease the workload of GPs [30].

Broadening the professional competencies of physiotherapists can also have a positive impact on patient care, as certain musculoskeletal diseases can be effectively treated by them. In some medical facilities in France, patients are referred directly to a physiotherapist, bypassing GPs [31]. Working in such a facility grants physiotherapists extended competencies. In consultation with a GP, it is possible to separate a group of patients requiring deeper specialist diagnostics from those requiring only conservative treatment. Importantly, both doctors and physiotherapists have positive opinions about the implementation of this model [32]. A similarly positive reception of this concept was also observed in New Zealand. Stotter et al. describe in their work the concept of the Advanced Practice Physiotherapist (APP), which aims to transfer part of the tasks usually performed by general practitioners in the care of patients with musculoskeletal pain to physiotherapists with extended clinical competencies [33]. The idea of introducing the APP role was generally well received by stakeholders, who perceived it as potentially beneficial for improving musculoskeletal care—although they highlighted the need to establish clear standards and conditions for implementation. See Table 1 for a summary of the roles and their competencies.

Rural communities frequently struggle with limited access to primary care physicians. The evidence reviewed above suggests that broadening the clinical duties of some already existing healthcare workers offers a practical solution for these local shortages. When these professionals take on independent consultations, manage chronic treatments, or perform initial musculoskeletal triage, local healthcare facilities become much more self-sufficient. Moreover, patients living in remote areas are less often forced to travel to distant secondary care centers for basic or preventive services, which is a major logistical benefit. Therefore, giving more responsibility to non-physician healthcare workers seems to be one of the most efficient ways to support rural medical care and ensure that patients receive timely treatment.

### 3.3. Transfer of Skills Between Professions

In healthcare models rooted in collaboration, different medical and paramedical professions work in a spirit of partnership. This improves treatment efficacy and shortens waiting times for medical services. On the other hand, implementation of the skill-mix model requires complex adjustments within the healthcare organization. The successful use of various specialists’ skills relies on an accurate process of categorizing health problems and aligning them with the available healthcare staff [34]. At the same time, there is an effort to reduce the administrative workload of doctors and nurses in many countries. This includes the implementation of medical assistants who take care of documentation or patient coordinators who improve healthcare organization [35,36].

Nursing is a profession that plays an increasingly significant role in modern healthcare systems. Nurses tend to take over responsibilities traditionally assigned to physicians, pharmacists, administrative employees, and other occupational groups. Studies have shown that nurses spend a substantial proportion of their working time on tasks outside core nursing activities, including approximately 32% on non-nursing tasks and about 12% on miscellaneous activities [37,38]. Evidence shows that the effectiveness of task-shifting models also depends on the attitudes of health workers. A review of primary studies found that unclear role boundaries, an increased workload, and a low sense of organizational support may limit acceptance of new forms of task redistribution [39].

In rural areas facing the crisis of medical deserts, adjusting professional roles through task sharing serves as a practical method to mitigate severe personnel shortages. Delegating documentation and coordination duties to medical assistants enables the limited local staff to shorten waiting times and focus on core clinical services. However, preventing an excessive workload and ensuring positive attitudes among health workers are critical to the effectiveness of such collaborative models in underserved regions.

### 3.4. Impact of Introducing Skill-Mix Methods

#### 3.4.1. Cost-Effectiveness

The costs of healthcare have been constantly rising for many years in various countries around the world. According to OECD data, health spending per capita in the U.S. has nearly tripled from 2000 to 2022 [40]. Similarly, Germany and the UK have also witnessed several-fold increases in healthcare costs. This is why healthcare systems are looking for new ways to increase cost-effectiveness, and the implementation of the skill-mix model may contribute to improved cost-effectiveness. The introduction of new professional roles within the healthcare workforce may, in some cases, be associated with a reduction in overall costs, especially after the new roles have adapted to the healthcare system [6]. In England, GP practice managers cite improved cost-effectiveness as one of the reasons for hiring workers in new professional roles [10]. Additionally, evidence from a recent scoping review confirms that an appropriate configuration of competencies within interdisciplinary teams is one of the key factors underpinning improvements in the quality of care [41,42]. However, cost reduction after introducing new roles has not always been observed, and in some cases, healthcare costs can even increase. Another idea aimed at reducing the cost of healthcare is to expand the competencies of certain health professions in order to allow them to take over some of the duties performed by professions that have higher wages. In Poland, nurses earn on average less than physicians, and their competencies are being expanded to include some previously physician-only duties [10,11].

#### 3.4.2. Quality of Care

When considering the introduction of the intersecting competencies model, it is important to assess its impact on the quality of patient care. Multiple examples from different health systems suggest that, when implemented correctly, the incorporation of skill-mix strategies may result in an improvement in the overall standard of medical care.

The introduction of new professional roles has been correlated with an increase in the quality of healthcare as well as increased patient satisfaction [6]. This observation is particularly evident within primary care, where patients often do not require specialized and complex care but rather are in need of more routine support. This is why new professional roles within primary care contribute to improving the continuity of patient care [43]. GP practice managers in England self-report that they are hiring additional staff precisely in an attempt to better meet their patients’ needs [8].

Expanding professional competencies has also been observed to have a positive effect on the quality and availability of healthcare for patients [31]. It has been proven that the participation of pharmacists in patient care resulted in better therapeutic outcomes as well as healthier lifestyles [24,28]. Thus, the overall quality of patient care is improved.

To summarize these findings, it is important to distinguish between the regulatory framework, practical experience, and empirical evidence. Legal regulations provide the necessary formal basis for skill-mix strategies, such as task shifting and the creation of new professional roles. However, legal changes alone do not necessarily lead to effective daily practice. Practical experience shows that medical facilities frequently face implementation barriers, for example, unclear role boundaries, training gaps, and a lack of organizational support. As noted earlier, these issues can cause resistance among existing staff and lead to increased work overload. Nevertheless, despite these practical difficulties, empirical evidence confirms that when skill-mix models are implemented properly, they bring clear advantages: clinical outcomes, increased access to care, and greater efficiency in medical practices.

A structured summary of the included sources is presented in Table 2.

## 4. Discussion

The examples analyzed in this review show that well-designed skill-mix strategies can be an efficient way to respond to health workforce shortages, especially in areas with limited access to healthcare. Global analyses indicate that task shifting and task sharing are expanding worldwide, with marked regional disparities and a growing involvement of new professional roles within health systems [44]. Many countries have already implemented different approaches to redistributing responsibilities between medical and paramedical professionals. Although the exact models and strategies vary, the ultimate goal is to use available human resources more effectively and more locally. Nowadays, the thriving skill-mix concept has the potential to play a significant role in combating medical deserts, which are globally responsible for increased mortality among people living in these regions as a result of poor access to specialists, diagnostic services and sufficient healthcare infrastructure. Rural and remote areas represent a significant part of underserved regions. The systematic review also showed that healthcare workers in rural areas are particularly vulnerable to work overload and reduced well-being, further underscoring the need to implement skill-mix strategies to stabilize local workforce capacity [45]. In the context of the skill-mix concept, healthcare systems can expand service coverage by optimizing the distribution of tasks among professionals, especially in underserved rural areas.

The skill-mix approach should focus on the individual needs of specific populations and local healthcare systems, ensuring that workforce models are tailored to the available resources, cultural context and other relevant factors in each region. Among many successful cases, such as France or Scotland, the key point was not to attract more specialists to rural areas but to reconsider which professionals can deliver specific services and relieve understaffed medical professionals of certain duties [16,17,31].

Certain strategies discussed in this paper may be relevant in the Polish context. Creating and implementing new professional roles, such as the advanced nurse practitioner or physician assistant, appears to be a practical and achievable strategy to reduce the burden on medical professionals by removing tasks unrelated to their professional scope [6]. Current initiatives such as POZ PLUS have also highlighted the need for a health coordinator in Poland [10,11]. However, due to the lack of training preparing individuals for this new role, the coordinator role is mainly performed by nurses. Telemedicine also remains an underused but highly promising tool, especially in the context of recent developments in artificial intelligence. However, successful integration will require not only technological infrastructure but also training and proper legal regulations regarding responsibilities and data protection. To better guide the transferability of international skill-mix models to the Polish healthcare system and its rural medical deserts, we synthesized the necessary systemic conditions into a structured framework. Table 3 outlines the key domains that policymakers must address to successfully adapt these solutions.

A major reason for implementing the skill-mix concept worldwide is the need to relieve physicians. Doctors are overburdened with workloads and responsibilities, often having to work overtime. In Japan, 11.8% of obstetrics/gynecology physicians in university hospitals work overtime for more than 1860 h per year [46]. This results in an extended time for delivering health services and poorer patient access to medical care. To prevent this situation and improve the effectiveness of healthcare systems, some of the responsibilities and simpler routine duties of medical doctors should be assumed by newly introduced professional roles. Expanding the competencies of other existing medical staff is yet another solution, which has been proven to be effective [32]. While physicians should remain key figures in primary care teams, studies show that their overall proportion within medical teams should be reduced [8]. By reducing their workload, medical doctors will be able to focus on more complex clinical cases, and they will assume a more coordinating role in patient care [10,43].

Anderson et al. emphasized that successful pharmacist integration requires clearly defined frameworks, trust from both patients and physicians, and precisely delineated competencies [30]. In the context of chronic diseases, pharmaceutical care leads to significant clinical benefits [24]. Świeczkowski et al. highlight that pharmacists, when properly trained and involved in therapy monitoring, can significantly improve patient compliance and quality of life, particularly in cardiovascular conditions [27]. The effectiveness of such interventions was also demonstrated in the Canadian RxEACH study, where pharmacist-led interventions resulted in a 21% relative reduction in estimated cardiovascular risk at 3 months (OR 0.79; 95% CI 0.68–0.91; *p* < 0.001) [25]. Similarly, research by Birand et al. showed that oncology pharmacists can impact not only treatment management but also patients’ beliefs about their medications, an essential factor in long-term therapies [28].

Nevertheless, despite the numerous advantages of expanding the competencies of healthcare personnel, certain risks must also be acknowledged. Nurses and paramedics, although equipped with extensive practical skills, do not always possess the same level of diagnostic expertise as physicians, which may increase the likelihood of clinical errors. One of the noted reasons for the ineffectiveness of new professional roles is a lack of mutual trust and clearly defined responsibilities within primary care teams. This organizational ambiguity hampers effective therapy management and may reduce the quality of care provided to patients [9,30]. The effective implementation of new professional roles within primary care teams requires not only task delegation but also appropriate professional training and clearly defined frameworks for collaboration and accountability within the team [20].

Moreover, many medical facilities, rather than hiring more staff, expect nurses to assume additional responsibilities [37,38]. Unfortunately, this practice may lead to nurses being overworked. This problem is especially noticeable in healthcare systems that face workforce shortages, where the introduction of further tasks is not followed by an increase in staff or adequate salary. Excessive workload and time pressure might also lead to professional burnout, elevated staff turnover, and a decline in overall work satisfaction. In the long term, this may weaken the entire healthcare system. Therefore, it is crucial to engage in proper planning and a clear definition of the roles and responsibilities of medical and allied health professions. According to implementation frameworks developed for healthcare systems, the effectiveness of task-shifting and task-sharing strategies also depends on adequate organizational preparation, an assessment of local needs, and continuous monitoring of care quality [47]. The experience from the British system is worth noting, where in 2015, the NHS initiated the integration of pharmacists into general practice in order to reduce the burden on doctors and improve pharmacotherapy management [29].

The skill-mix concept clearly requires further research. While existing data show several promising strategies, there is still a lack of reliable evidence on how these solutions function in regions with limited resources and uneven distribution of healthcare, such as medical deserts. Future research should focus not only on describing individual models but also on evaluating their implementation in different settings. This need is particularly urgent in the Polish context, as it would allow for a meaningful, evidence-based assessment of whether and how skill-mix solutions lead to measurable improvements. Moreover, comparative studies, preferably with countries in Central and Eastern Europe that share similar demographic trends, historical background and healthcare system limitations, could bring valuable insights.

The main limitation of this review is the lack of access to consistent, comparable data across different countries, especially when it comes to long-term outcomes and the effectiveness of specific skill-mix strategies. Many reports are either unavailable in English, not publicly obtainable, or lack standardized indicators, which makes it difficult to draw clear conclusions or compare results across different healthcare systems. This also means that some potentially valuable local initiatives may have been missed due to a lack of proper documentation or evaluation. Moreover, healthcare systems in different countries operate in their own context, shaped by priorities and resources. A strategy that works well in one country might not be effective in another—not because the idea is wrong, but because of the difference in the level of healthcare funding, infrastructure, or even the historical structure of the healthcare system. Moreover, reported outcomes varied considerably across studies and were not uniformly defined, which limited direct comparison between countries.

Additionally, it must be noted that the included publications often focused solely on the clinical and organizational outcomes of introducing skill-mix solutions. The consideration of the influence of professional regulators, such as medical or nursing unions and chambers, was not deeply analyzed in this review and remains a vital area for future research.

Furthermore, a potential publication bias must be acknowledged, as successful skill-mix implementations are substantially more likely to be documented and published than failed pilot programs. Consequently, the actual challenges of implementation may be underestimated in the available literature.

## 5. Conclusions

In this review, we identify three main areas of the intersecting competencies concept: the introduction of new professional roles into the healthcare system, the expansion of competencies of existing medical professions, and the transfer of skills between professions. Although no single model of implementation is universally effective, adopting skill-mix solutions is no longer optional but appears necessary, as the efficient allocation of patient care tasks may contribute to overall improvements in care delivery and the stability of the healthcare system.

Based on the evidence presented in the included studies, the implementation of the skill-mix concept is associated with both positive and negative outcomes. When properly introduced, it may offer several advantages—including reducing the administrative and clinical burden on doctors, supporting the quality of patient care, and increasing patient satisfaction. Skill-mix strategies may improve access to healthcare services for patients. The observed effects appear to be context-dependent and vary across healthcare systems.

Furthermore, appropriate implementation of the skill-mix concept—both through the creation of new professional roles and the expansion of competencies of existing professions—may contribute to reduced healthcare costs and improved operational efficiency of healthcare facilities. However, these effects are typically observed after an adaptation period and depend on organizational and institutional conditions, indicating that the system requires time to adjust to newly introduced roles and workflows.

Ultimately, the purpose of this analysis is to support discussion on evidence-based systemic changes and to guide policymakers, particularly in regions that are struggling with uneven healthcare availability. The skill-mix methods identified in this review, which are already being implemented in various healthcare settings, may contribute to addressing challenges related to the phenomenon of medical deserts and the growing need for healthcare. However, effective implementation necessitates a permanent update of professional regulations across different healthcare systems alongside the simultaneous creation of interdisciplinary educational standards. Such a comprehensive approach may help ensure that healthcare workforce optimization will lead to an overall more resilient and patient-centered healthcare system. The strength of these conclusions is limited by the heterogeneity of the included studies and the variability in reported outcomes.

## Figures and Tables

**Table 1:** Overview of selected professional groups participating in skill-mix initiatives, including their main competencies and documented benefits for healthcare delivery.

Profession	Scope of Competencies	Benefits
Specialist nurses/registered nurses	Diagnosis, treatment, prescribing medications, and care for chronically ill patients	Faster access to care; reduced burden on physicians
Nurses	Administrative duties (medical documentation, patient coordination)	Reduced administrative workload for physicians
Advanced nurse practitioner (ANP)	Diagnosis, treatment, prescribing medications, and patient education	Improved access to care; reduced physician workload; enhanced clinical outcomes
Nurse aides (NAs, Taiwan)	Support for nurses in daily duties	More nursing time dedicated to patient care; cost reduction
Clinical pharmacists	Medication reviews, follow-up consultations, and patient education	Fewer medication errors; improved patient adherence; reduced burden on physicians
Pharmacists in primary care (Great Britain)	Medication reviews, monitoring of chronic treatments, and patient consultations	Improved pharmacological care; reduced physician workload
Paramedics in primary care	Support in managing chronically ill patients	Relief for primary care teams; improved access to care
Physiotherapists	Diagnosis of musculoskeletal disorders, issuing sick leave certificates, and prescribing medications	Faster access to rehabilitation; reduced burden on general practitioners
Doctors’ assistants	Support in diagnosis; conducting parts of patient consultations	Increased appointment availability; improved organization of physicians’ work
Healthcare coordinators	Appointment scheduling, patient record management, initial qualification for diagnostic tests	Streamlined administration; improved care pathway coordination
Expanded function dental auxiliaries (EFDAs)	Extended dental assistant duties (e.g., applying dental sealant)	Increased number of dental procedures; reduced workload for dentists
Dental assistants (Scotland, “Childsmile” program)	Oral health prevention, dietary advice, and fluoride application	Improved preventive care; reduced burden on dentists

**Table 2:** Summary of included sources and key characteristics of analyzed skill-mix interventions.

Author (Year)	Country	Setting	Cadre(s) Involved	Skill-Mix Strategy	Regulatory/Financing Context	Outcomes Reported	Study Type
Tsiachristas et al. (2015) [6]	High-income countries (mostly the UK, USA, Netherlands, and Canada)	Various settings, including primary, ambulatory, acute, and long-term care, often focusing on chronic diseases	Specialist nurses (SNs) and advanced nurse practitioners (ANPs) compared to traditional doctors	Task substitution, involving extended roles (SNs) or new roles (ANPs) for tasks like follow-ups, case management, and first consultations	Driven by rising healthcare expenditure and the need for cost-containment and efficiency improvements	Access, patient information, satisfaction, clinical outcomes, quality of life, quality of care, mortality, utilization, and costs	Systematic literature review comprising 41 studies (predominantly RCTs)
NHS England and NHS Improvement (2019) [7]	England (UK)	Primary care/general practice (specifically primary care networks—PCNs)	Clinical pharmacists, social prescribing link workers, physician associates, physiotherapists, and paramedics	Expanding multidisciplinary teams by recruiting up to 20,000 new full-time equivalent (FTE) posts across these five roles to address workforce shortages in general practice	Implementation of the “Additional Roles Reimbursement Scheme” under the Network Contract DES framework. It provides 70–100% salary reimbursement strictly for demonstrably new (additional) staff, preventing funds from being used to fill existing vacancies	N/A (This is not an empirical research study, so no clinical or economic outcomes are measured)	Official policy guidance/implementation framework
Gibson et al. (2022) [8]	England (UK)	Primary care/GP practices	Advanced nurse practitioners, specialist nurses, healthcare assistants, physician associates, paramedics, and pharmacists	Employing diverse non-medical staff to match patient needs, increase appointment availability, and release GP time	Driven by GP shortages and increasing patient complexity; financially supported by the Additional Roles Reimbursement Scheme (ARRS) via Primary Care Networks (PCNs)	Practice managers’ motivations for hiring, external funding utilization, and ideal vs. current workforce preferences	Cross-sectional survey
Nelson et al. (2019) [9]	England (UK)	Primary care/general practice	Advanced practitioners (APs), physician associates (PAs), and practice pharmacists (PPs)	Introducing non-medical roles to work alongside GPs to fill workforce gaps, cope with demand, and release GP time	Driven by GP shortages; schemes funded via local CCGs and Health Education England (HEE); lack of statutory regulation for PAs is noted as a barrier to integration	Qualitative implementation experiences: role definition, professional boundaries, risk management, training-practice gaps, managing expectations, and role sustainability	Qualitative comparative study (interviews and focus groups)
Kowalska-Bobko et al. (2020) [10]	Poland (with references to selected EU countries)	Various settings, including primary care, hospitals, emergency medical services, and rehabilitation	Nurses, midwives, paramedics, physiotherapists, and medical coordinators	Role expansion and task substitution (e.g., independent prescribing for nurses, independent visits for physiotherapists, and extended duties for paramedics)	Driven by severe medical staff shortages and demographic changes; enabled by new national legislation expanding professional rights and the introduction of coordinated care programs	N/A (descriptive review; no empirical clinical or economic outcomes measured)	Review article/policy analysis
Narodowy Fundusz Zdrowia (NFZ) (2024) [11]	Poland	Primary care (POZ)	Doctors, nurses, care coordinators, managers, dietitians, and other medical/non-medical staff	Upskilling and training multidisciplinary teams across 1500 facilities to effectively implement and manage coordinated care	EU-funded project (European Social Fund Plus) executed by the Ministry of Health and NFZ (~121.6M PLN) to integrate healthcare and improve access, especially in rural areas	N/A (this is a project description, not an empirical study; it outlines expected results like new training modules, guidelines, and a maturity assessment tool)	Official project description/implementation framework
Reeves et al. (2020) [12]	Switzerland	Primary palliative home care	General practitioners (GPs), nurses, and family members	Informal role shifting where nurses and family members assume care coordination and decision-making responsibilities, often substituting for unavailable GPs	Absence of standardized care coordination practices in Switzerland; lack of formal financial or psychosocial reimbursement for family members undertaking coordination tasks	Qualitative experiences, including role ambiguity, interprofessional conflicts, overburdened family members, and coping strategies like clear communication and team stability	Qualitative study (semi-structured interviews)
Wanyonyi et al. (2015) [13]	England (UK)	Primary dental care (state-funded National Health Service)	Dentists and mid-level dental providers/dental care professionals (DCPs), specifically dental therapists and hygienists	Task sharing and delegation of diagnostic tasks, preventive care (e.g., fluoride varnish), and routine restorative treatments from dentists to dental therapists	Driven by cost-containment and changing population oral health needs; facilitated by “Direct Access” regulations allowing patients to see DCPs without a dentist referral, though NHS funding mechanisms remain a barrier	Estimated clinical time, whole-time equivalent (WTE) workforce numbers, and salary costs across four different delegation scenarios	Operational research (supply and demand simulation modeling/scenario testing)
Conrad et al. (2010) [14]	USA (Oregon)	Primary dental care/general dental practices	General dentists, dental assistants, and dental hygienists	Utilizing dental auxiliaries (assistants and hygienists) alongside dentists to maximize practice productivity and output	Driven by rising dental expenditures, price inflation, and access gaps; analyzes the impact of practice ownership (owner vs. non-owner) and payer mix (% Medicaid) on efficiency	Dentist productivity (measured as patient visits per week) as a function of labor inputs (dentist hours, number of auxiliaries) and capital inputs (number of operatories)	Cross-sectional survey and economic analysis (OLS regression and path analysis)
Ivanoff et al. (2023) [15]	Bulgaria	Dental care/dental practices	Dentists and dental assistants/expanded function dental auxiliaries (EFDAs)	Expanding the skillset of dental assistants to perform specific expanded duties (e.g., restorative procedures) without the personal supervision of a dentist	Driven by severe regional disparities in dentist distribution, demographic crisis, and high unmet oral health needs among rural and minority populations; currently restricted by labor laws requiring “personal supervision” by a dentist	Perceptions of dentists and assistants regarding task delegation, expected practice efficiency, required training, and patient trust	Cross-sectional survey
Childsmile/NHS Scotland (N/A) [16]	Scotland (UK)	Public health/primary dental care	NHS dentists, health professionals, educators, and community/voluntary sector workers	Multi-sector collaboration to promote preventive oral health and ensure access to dental services for children	State-supported (NHS) public health program focused on reducing oral health inequalities for all children, regardless of income or background	N/A (descriptive program overview)	Program description
Gnich et al. (2014) [17]	Scotland (UK)	General dental practice	General dental practitioners (GDPs) and extended-duty dental nurses (EDDNs)	Role supplementation: EDDNs delivering clinical preventive care (e.g., fluoride varnish application, dietary and oral hygiene advice) traditionally undertaken by GDPs	Driven by the national “Childsmile” program to tackle child oral health inequalities; facilitated by regulatory changes extending dental nurses’ duties, and supported by financial incentives (fees for preventive care)	EDDNs’ role satisfaction, perceived utility of training, self-reported frequency of preventive delivery, and behavioral mediators (motivation, skills, barriers/facilitators)	Cross-sectional postal survey
Parliament of Poland (2014) [18]	Poland	National healthcare system	Nurses and midwives	Legal authorization for qualified nurses and midwives to independently prescribe certain medications and medical devices and issue diagnostic referrals	A national legislative act amending healthcare laws to integrate nurse/midwife prescriptions into the public reimbursement system (NFZ)	N/A (this is a legal document establishing new rights and restrictions and prescribing procedures, not an empirical study reporting outcomes)	Legislation/legal act
Minister of Health, Poland (2018) [19]	Poland	National healthcare system (specifically, outpatient and home-based medical rehabilitation)	Physiotherapists and physicians	Expanding physiotherapists’ autonomy to independently conduct “physiotherapy visits,” plan treatments, and modify physician referrals (requiring consultation only for certain specialists)	A national regulation standardizing the “physiotherapist” title and expanding their independent competencies within guaranteed medical rehabilitation benefits	N/A (Legal regulation establishing new physiotherapy procedures, not an empirical study)	Legislation/legal regulation
Lovink et al. (2018) [20]	The Netherlands	Primary healthcare (general practices and community care) focusing on older people	General practitioners (GPs), nurse practitioners (NPs), physician assistants (PAs), and registered nurses (RNs)	Introducing NPs, PAs, and RNs as physician substitutes or supplements to provide routine consultations and proactive healthcare (e.g., geriatric assessments, case management) in order to reduce GP workload	Driven by “ageing in place” policies, growing elderly populations, and heavy GP workload; facilitated by legislation allowing NPs and PAs to perform medical tasks and independently prescribe drugs	Qualitative perceptions on task division, responsibilities, collaboration barriers/facilitators, and perceived impact (e.g., improved care quality and shifting the GP role to focus on complex cases)	Qualitative study (focus groups and individual interviews)
van Erp et al. (2021) [21]	International (included studies from the USA, UK, Canada, and New Zealand)	Primary care/community care (“primary care plus”)	Nurse Practitioners (NPs) and Physician Assistants (PAs) (however, all 15 included studies ultimately focused only on NPs	Substitution of care (“primary care plus”)—shifting specialist medical care and consultations from hospitals to primary/community care settings, delivered by NPs	Driven by rising healthcare costs, aging populations, and multimorbidity, structural funding and reimbursement are cited as key barriers to implementation	Quality of care (health status, mortality, patient satisfaction), hospital admission/referral rates, costs, and facilitators/barriers	Systematic review (15 studies)
Huang et al. (2011) [22]	Taiwan	Hospital acute care/medical wards	Registered nurses (RNs) and nurse aides (NAs)	Introducing Nas to assist with daily patient care, which shifts RNs’ duties towards delegating tasks, educating, and supervising the Nas	Driven by severe global nursing shortages, cost-containment pressures, and infection control concerns highlighted during the 2003 SARS outbreak; initiated by Taiwan’s Health Department projects	Changes in nurses’ perceived role functions (independent, dependent, and interdependent), job/role satisfaction, and perceived patient care quality	Cross-sectional survey
Paier-Abuzahra et al. (2024) [23]	International (studies mostly from the UK, Netherlands, USA, and Canada)	Primary care/general practice	Primary care physicians (PCPs) and various nursing roles (registered nurses, nurse practitioners, advanced nurse practitioners)	Task-shifting (delegation or substitution) of medical activities (such as first contact, patient assessment, prescribing, and ongoing chronic care) from PCPs to nurses	Driven by global shortages and maldistribution of medical doctors across OECD countries. Implementation requires organizational flexibility and adjustments to existing hierarchies and legislation, as well as higher remuneration for nurses assuming greater responsibilities	Patient-relevant outcomes (mortality, hospital admissions, patient satisfaction, quality of life), clinical surrogate outcomes (e.g., blood pressure, HbA1c), and health services-related outcomes (e.g., consultation length, emergency attendances, prescriptions)	Overview of systematic reviews (Umbrella review)
Santschi et al. (2011) [24]	International (mostly North America, along with South America, Asia, Europe, and Australia)	Primary care/outpatient clinics and community pharmacies	Pharmacists, primary care physicians, and nurses	Pharmacist-directed or collaborative care for cardiovascular disease (CVD) risk management, including medication management, patient education, patient-reminder systems, and providing feedback/recommendations to physicians	Driven by rapidly rising healthcare costs, suboptimal CVD risk control in the population, and patients’ difficulties in accessing primary care physicians, which promotes greater use of community-based models of care	Clinical outcomes, including changes in systolic and diastolic blood pressure, total cholesterol, low-density lipoprotein cholesterol (LDL-C), and smoking rates	Systematic review and meta-analysis of randomized controlled trials (30 RCTs)
Tsuyuki et al. (2016) [25]	Canada (Alberta)	Community pharmacies	Community pharmacists and family physicians	Expanding the scope of practice for community pharmacists to include proactive case finding, cardiovascular risk assessment, independent prescribing of medications, and ordering laboratory tests	Enabled by legislation in Alberta granting pharmacists an advanced scope of practice (independent prescribing authority and ability to order/interpret lab tests); supported by a provincial remuneration program (Alberta Health) that reimburses pharmacists for medication management services	Estimated risk for future cardiovascular events, as well as changes in individual risk factors such as systolic and diastolic blood pressure, LDL cholesterol (LDL-C), glycemic control (HbA1c), and smoking cessation	Multicenter randomized controlled trial (RCT)
Chaudhri et al. (2023) [26]	International (included 27 studies from 10 countries, with the majority from the USA)	Primary care	General practitioners (GPs)/primary care physicians and pharmacists	Interprofessional, bidirectional collaboration (verbal or written communication, often co-located) involving patient education, medication regimen assessment, adherence monitoring, and physical assessments	Driven by the rising global prevalence of cardiovascular disease, treatment gaps in primary care, maldistribution of physicians, and high costs of physician-centered care, highlighting the need to better utilize highly accessible pharmacists	Changes in cardiovascular risk factors (systolic and diastolic blood pressure, total cholesterol, LDL, HDL, HbA1c, BMI, and smoking cessation rates) and healthcare costs (visit and medication charges)	Systematic review and meta-analysis of randomized controlled trials (27 RCTs)
Swieczkowski et al. (2016) [27]	Poland	Community pharmacies and hospital wards/outpatient cardiovascular care	Pharmacists and physicians (general practitioners, cardiologists)	Integrating pharmacists into multidisciplinary care teams to provide advanced pharmaceutical services (pharmaceutical care), including medication reviews, adherence monitoring, patient education, and identifying drug-related problems	Currently limited in Poland, as pharmacies primarily focus on drug dispensing, significant barriers include a lack of public reimbursement for advanced pharmaceutical services and a lack of integrated communication/IT tools between doctors and pharmacists. However, legislative changes are being discussed to expand the pharmacist’s clinical role	As a review, it synthesizes evidence from other studies on health-related quality of life, medication adherence, clinical cardiovascular risk factors (blood pressure, lipid profile, and HbA1c), and reductions in drug-related problems	State-of-the-art review/special article
Birand et al. (2019) [28]	Northern Cyprus	Hospital/oncology department	Oncology pharmacists (alongside oncology nurses and physicians)	Integration of clinical oncology pharmacists to provide direct, face-to-face patient education and counseling (regarding treatment plans, management of side effects, and rational drug use) in order to improve medication beliefs and adherence	Cancer medications are state-funded and excluded from community pharmacies; the intervention introduces previously absent clinical oncology pharmacist roles	Patient medication adherence (measured by the Morisky Green Levine Test) and patients’ beliefs about medicines, specifically the necessity-concern balance, general overuse, and general harm (measured by the Beliefs about Medicines Questionnaire)	Interventional prospective study
NHS England (2015) [29]	England (UK)	General practice/primary care	General practitioners (GPs) and clinical pharmacists	Integrating clinical pharmacists into GP practice multidisciplinary teams to manage long-term conditions, advise on multiple medications, provide clinical treatment advice, and alleviate GP workload	Driven by severe GP workforce shortages, escalating patient demand, and unprecedented workload. Enabled by a £31m national pilot funding scheme by NHS England as part of the “GP Workforce 10 Point Plan”	N/A (descriptive program overview)	News release (policy and practice resource)
Anderson et al. (2019) [30]	International (mostly UK/England, but includes studies from Australia, Canada, Iceland, etc.)	General practice/primary care	Clinical pharmacists and general practitioners (GPs)	Integrating clinical pharmacists into general practice to conduct medication reviews, solve medication-related problems, act as independent prescribers, and reduce GP workload	Driven by an aging population, rising demand for primary care, and severe GP workforce shortages. Supported by the NHS England national pilot scheme (backed by up to £100m in funding) as part of the “General Practice Forward View” to expand the primary care workforce	Stakeholder perspectives (patients, GPs, and pharmacists), implementation barriers and facilitators, and key mechanisms for success (patient trust, GP confidence, pharmacist capability, funding, and flexible delivery models)	Realist review (43 papers relating to 38 studies)
Kechichian et al. (2024) [31]	France	Primary care	Physiotherapists (PTs) and family physicians (FPs)	First-contact physiotherapy (FCP) allows PTs to independently assess low back pain, prescribe medication, and issue sick leave	Driven by physician shortages; enabled by French “Cooperation Protocols” which delegate medical acts to non-medical professionals	Disability, pain, healthcare resource use, wait times, and satisfaction, among others	Cluster randomized controlled trial (RCT)
Kechichian et al. (2022) [32]	France	Primary care/multidisciplinary healthcare centers	Physiotherapists (PTs) and family physicians (FPs)	Task sharing and shifting allow PTs to act as first-contact practitioners to diagnose acute low back pain, prescribe analgesic medication, and issue sick leave	Driven by primary care saturation and workforce shortages; enabled by a new legislative text allowing protocol-based care delegation	Acceptability of the model, perceptions of PTs’ competencies, and perceived barriers/facilitators to implementation	Cross-sectional survey
Stotter et al. (2024) [33]	New Zealand	Primary healthcare	Physiotherapists (including Advanced Practice Physiotherapists), physicians (general practitioners and medical specialists), and ACC case managers.	Introducing Advanced Practice Physiotherapists (APPs) to manage complex musculoskeletal conditions, triage referrals, provide second opinions, and undertake other related activities	Driven by escalating healthcare costs, specialist workforce shortages, and inefficient primary/secondary care interfaces; enabled by the 2021 Physiotherapy Board regulation of the APP scope	Stakeholders’ perceptions, implementation barriers/enablers, potential impact on patient pathways, and career progression	Qualitative interview study
Spooner et al. (2022) [34]	England (UK)	General practice/primary care	General practitioners (GPs), diverse non-GP workforce (e.g., advanced clinical practitioners, advanced nurse practitioners, clinical pharmacists, physician associates, paramedics, physiotherapists), and reception staff	Redistributing unfiltered patient problems from GPs to a diverse non-GP workforce through complex organizational processes, including categorizing patient problems, defining practitioner skillsets, and flexibly matching patients to the appropriate practitioner	Driven by a severe GP workforce crisis and increasing demand for care, enabled by government health policy and subsidized funding for employing diverse practitioners across Primary Care Networks under new contracts	Processes of skill-mix implementation (categorization and matching), mechanisms of flexibility and escalation, and patient experiences with these adaptations	Qualitative case study (including interviews, observations, and focus groups)
Supreme Audit Office of Poland (NIK) (2021) [35]	Poland	Ambulatory healthcare: specifically POZ (primary healthcare) and AOS (ambulatory specialist care)	Doctors, nurses, midwives, medical assistants, and administrative staff.	Evaluating task-shifting: delegating e-documents to medical assistants and independent consultations to nurses/midwives	Driven by staff shortages and heavy administrative burdens. Supported by the 2018–2019 legal changes enabling delegation	Severe underutilization of skill mix. Doctors spent 28–43% of visit time on administration, 86% of facilities did not use medical assistants, and 91% burdened nurses with clerical tasks.	National audit report
Lack et al. (2019) [36]	Canada	Acute care surgery (ACS) within an academic tertiary hospital	Physician assistants (PAs), physicians (staff surgeons and surgical residents), and allied health providers	Integrating a PA into the ACS team to manage daily ward issues, patient encounters, and multidisciplinary meetings, which ensures care continuity and allows residents more operating time	Driven by resident shortages; PAs in Ontario are unregulated and work under medical directives; a major barrier is the lack of sustainable funding, relying on temporary annual contracts	Quantitative metrics of PA involvement (encounters, consults, meetings), plus qualitative feedback on resident/staff satisfaction and team efficiency	Prospective descriptive study
Grosso et al. (2021) [37]	Italy	Hospitals, community settings, and residential care (nursing homes)	Registered nurses (RNs)	An unintended skill-mix consequence where nurses routinely perform “non-nursing tasks” outside their scope, highlighting the need for better task delegation to support staff	Driven by cost-cutting measures, spending reviews, and systemic human resource shortages, which force nurses to compensate for missing support staff	The prevalence and types of non-nursing tasks and factors influencing their occurrence (e.g., resource adequacy and clinical setting)	Cross-sectional survey study
Michel et al. (2021) [38]	Switzerland	Internal medicine ward in a university hospital	Registered nurses (RNs) and auxiliary nurses (ANs)	Evaluation of RN and AN task distribution, highlighting suboptimal use of RNs’ scope and recommending the reallocation of non-clinical tasks to increase direct patient care	Driven by complex care and limited resources. Switzerland lacks national nurse-to-patient ratios, and nurses’ clinical autonomy lacks legal recognition in federal health insurance	Time allocated to various practice dimensions, revealing that RNs spend a limited proportion of their work time directly with patients	Observational descriptive study (time and motion analysis)
Coales et al. (2023) [39]	Low- and middle-income countries (LMICs), including regions such as sub-Saharan Africa, South Asia, and South East Asia	Diverse healthcare environments, predominantly primary healthcare and community settings, but also including secondary and tertiary care	Specialist health workers (SHWs, e.g., doctors, senior nurses) and non-specialist health workers (NSHWs, including lay health workers)	Task shifting from scarce SHWs to larger cadres of NSHWs, with SHWs taking on supervisory and training roles to expand service capacity	Driven by SHW shortages and rising healthcare demand; success relies on secure funding for resources and clear governance policies to prevent exploitative, unregulated practices	Health workers’ perspectives on task shifting, focusing on the workplace environment, access to resources, and alignment with personal values and emotional resilience	Qualitative evidence synthesis (QES)
Organisation for Economic Co-operation and Development (OECD) (N/A) [40]	International/OECD member countries	Macroeconomic health policy/National health systems	N/A (Macro-level data)	N/A (Provides the economic rationale for implementing cost-effective skill-mix interventions, rather than describing a specific clinical strategy)	Defines “health spending” as the final consumption of healthcare goods and services, tracked as a percentage of GDP; skyrocketing costs drive the global need for workforce reorganization	Macroeconomic indicators such as health expenditure and financing	Statistical database/policy definition
Parliament of Poland (2022) [41]	Poland	Healthcare entities	Diverse health workforce (doctors, nurses, midwives, allied health professionals like physiotherapists and pharmacists, medical caregivers, and other basic activity staff)	N/A (this is a legal act establishing a statutory minimum wage matrix based on education and specialization levels, rather than a clinical task-shifting intervention)	A national law determines the lowest basic salary for healthcare workers by multiplying a specific work coefficient by the average national wage, effectively linking guaranteed pay levels to required qualifications	N/A (legal text providing a salary classification table for specific professional groups and mechanisms for wage increases, not an empirical study)	Legislation/legal act
Organisation for Economic Co-operation and Development (OECD) (2025) [42]	Poland	Macroeconomic health policy/national healthcare system	The entire national healthcare workforce (including doctors, nurses, midwives, dentists, pharmacists, paramedics, healthcare assistants, recovery assistants, care coordinators, and long-term care workers)	Recommends task-shifting by training healthcare assistants for administrative duties, expanding nursing roles to free up doctors, and utilizing “recovery assistants” in mental health	Driven by severe shortages of healthcare workers, an aging workforce, and rising demand due to an aging population, public healthcare spending is historically low but is planned to increase to 7% of GDP by 2027	Macro-level evaluations of health system efficiency, workforce shortages, and projections of future healthcare staff demand	Economic survey/policy report
Lovink et al. (2019) [43]	The Netherlands	Nursing homes	Elderly care physicians (ECPs), nurse practitioners (NPs), physician assistants (PAs), and registered nurses (RNs)	Substituting ECPs with NPs, PAs, or RNs to perform medical tasks previously reserved for physicians (e.g., patient intakes, medical rounds, prescribing medication) in order to reduce physician workload	Driven by an aging population, rising chronic disease rates, and a shortage of ECPs; implementation is sometimes facilitated by government grants for training	Variations in task organization, factors influencing success (e.g., vision, acceptance), and positive impacts on care quality and ECPs’ coordinating roles	Qualitative study (focus group interviews)

**Table 3:** Transferability framework for implementing skill-mix strategies in rural medical deserts.

Transferability Domain	Key System Conditions
Governance and regulation	Clear legal boundaries for new competencies and official recognition of new roles (such as independent prescribing).
Workforce supply	Sufficient local availability of allied health professionals to take over delegated tasks from doctors.
Payment arrangements	Dedicated structural funding (such as POZ PLUS) that secures salaries without burdening local facility budgets.
Training capacity	Certified interdisciplinary educational standards and mentorship programs for expanded clinical duties.
Professional acceptance	Building mutual trust and demonstrating that task-shifting effectively relieves doctors rather than competing with them.
Rural infrastructure	Adequate physical workspaces and secure IT systems (telemedicine) to enable safe task sharing in remote areas.

## Data Availability

The original contributions presented in this study are included in the article.

## Appendix A. Gray Literature Search Strategy

**Source/Organization**	**Website Domains**	**Search Terms**	**Dates Searched**	**Selection Criteria**
World Health Organization (WHO)	who.int	“skill mix”, “task shifting”, “health workforce”	February 2025–March 2025	Foundational frameworks and global guidelines defining skill-mix concepts and task-shifting principles.
National Health Service (NHS England)	england.nhs.uk	“Additional Roles Reimbursement Scheme”, “skill mix”	October 2025–November 2025	Official guidance documents, reimbursement policies (e.g., ARRS), and systemic blueprints for integrating new roles in primary care.
National Health Service Scotland (NHS Scotland)	nhs.scot	“Childsmile”	October 2025–November 2025	Institutional reports and evaluations of national prevention programs utilizing extended professional scopes (e.g., dental nurses).
National Health Fund (Narodowy Fundusz Zdrowia—NFZ)	nfz.gov.pl	“opieka koordynowana w POZ” [coordinated primary healthcare]	October 2025–November 2025	Official programmatic guidelines and structural frameworks detailing the implementation of coordinated care models in Polish primary healthcare.
Polish Legislative Bodies and Official Legal Databases, including the Polish Ministry of Health (Ministerstwo Zdrowia)	sejm.gov.pl, isap.sejm.gov.pl, dziennikustaw.gov.pl	“ustawa o zawodach pielęgniarki i położnej” [act on the professions of nurse and midwife], “rehabilitacja lecznicza” [medical rehabilitation], “minimalne wynagrodzenie” [minimum salary]	October 2025–November 2025	Binding national laws and ministerial regulations that explicitly authorize new scopes of practice (e.g., prescribing rights and rehabilitation tasks) and establish statutory remuneration tiers for the healthcare workforce.
Supreme Audit Office of Poland (Najwyższa Izba Kontroli—NIK)	nik.gov.pl	“organizacja pracy” [work organization], “ambulatoryjna opieka specjalistyczna” [outpatient specialist care]	October 2025–November 2025	Formal state audit reports providing empirical assessments of healthcare organizations, task allocation, and workforce inefficiencies.
Organisation for Economic Co-operation and Development (OECD)	oecd.org	“health spending”	October 2025–November 2025	International economic comparative health data and reports on healthcare system performance.
Ministry of Health, Labour and Welfare (Japan)	mhlw.go.jp	“working conditions of doctors”, “overtime”	October 2025–November 2025	National surveys on healthcare working conditions and workforce policies.
